# Interaction of *Clostridium perfringens* Epsilon Toxin with the Plasma Membrane: The Role of Amino Acids Y42, Y43 and H162

**DOI:** 10.3390/toxins14110757

**Published:** 2022-11-03

**Authors:** Skye Marshall, Beth McGill, Helen Morcrette, C. Peter Winlove, Catalin Chimerel, Peter G. Petrov, Monika Bokori-Brown

**Affiliations:** 1 Department of Physics and Astronomy, University of Exeter, Stocker Road, Exeter EX4 4QL, UK; 2 College of Life and Environmental Sciences, University of Exeter, Stocker Road, Exeter EX4 4QD, UK; 3 Automation Department, Faculty of Electrical Engineering and Computer Science, Transilvania University of Brasov, 500036 Brasov, Romania; 4 Living Systems Institute, University of Exeter, Stocker Road, Exeter EX4 4QD, UK

**Keywords:** *Clostridium perfringens* epsilon toxin, pore forming toxin, aerolysin family, multiple sclerosis, plasma membrane, artificial lipid bilayers, myelin and lymphocyte protein (MAL), Chinese hamster ovary cells, red blood cells

## Abstract

*Clostridium perfringens* epsilon toxin (Etx) is a pore forming toxin that causes enterotoxaemia in ruminants and may be a cause of multiple sclerosis in humans. To date, most in vitro studies of Etx have used the Madin-Darby canine kidney (MDCK) cell line. However, studies using Chinese hamster ovary (CHO) cells engineered to express the putative Etx receptor, myelin and lymphocyte protein (MAL), suggest that amino acids important for Etx activity differ between species. In this study, we investigated the role of amino acids Y42, Y43 and H162, previously identified as important in Etx activity towards MDCK cells, in Etx activity towards CHO-human MAL (CHO-hMAL) cells, human red blood cells (hRBCs) and synthetic bilayers using site-directed mutants of Etx. We show that in CHO-hMAL cells Y42 is critical for Etx binding and not Y43 as in MDCK cells, indicating that surface exposed tyrosine residues in the receptor binding domain of Etx impact efficiency of cell binding to MAL-expressing cells in a species-specific manner. We also show that Etx mutant H162A was unable to lyse CHO-hMAL cells, lysed hRBCs, whilst it was able to form pores in synthetic bilayers, providing evidence of the complexity of Etx pore formation in different lipid environments.

## 1. Introduction

Epsilon toxin (Etx) is produced by *Clostridium perfringens* type B and D strains and plays a key role in enterotoxaemia, a severe disease of domestic ruminants that mainly affects sheep. More recently, a growing body of evidence has implicated Etx as a trigger for the development of multiple sclerosis (MS) in humans, a neurological disease of young adults [1,2,3,4].

The 50% lethal dose (LD_50_) of Etx in mice after intravenous injection is 100 ng kg^−1^, equivalent to 7 mg in a 70 kg human, making Etx the third most potent bacterial toxin known [5,6]. For this reason, Etx is classified as a category B biological agent by the U.S. public health system, creating the need for more research in Etx mode of action in human model systems.

Etx typically acts by forming pores in the target cell membrane in a multi-step process that is proposed to involve binding, oligomerisation and pore formation. Like most pore forming toxins (PFTs), Etx is released from the bacterium as an inactive, water-soluble monomer, called protoxin (P-Etx). The P-Etx is then activated by proteolytic cleavage of the carboxy-terminal peptide (CTP) by digestive proteases of the host, such as trypsin, or by *Clostridium perfringens* λ protease [7,8]. Both P-Etx and activated Etx can bind to cells but only activated toxin with CTP removed can oligomerize and form pores in target cell membranes [9,10,11]. Pore formation disrupts the membrane integrity by inducing a rapid loss of intracellular K^+^ and an increase in Cl^−^ and Na^+^, with a subsequent increase in Ca^2+^ [12], which typically leads to cell death.

In the past few decades many cell lines have been tested for their sensitivity to Etx but only a few have been identified to be sensitive, such as kidney cell lines of dog, mouse and human origin [13], the mouse lung endothelial cell line 1G11 [14], lymphocytes [15], human erythrocytes [16,17], and cells originating from the thyroid [18] and central nervous system [19]. To date, most in vitro studies of Etx use the Madin-Darby canine kidney (MDCK) cells, a dog kidney cell line of epithelial origin, as this cell line shows high sensitivity to Etx [20,21,22,23]. However, more recently Chinese hamster ovary (CHO) cells engineered to express the putative Etx receptor myelin and lymphocyte protein (MAL) have also been shown to be highly sensitive to Etx [2,24].

MAL is an integral membrane protein constituent of lipid rafts with a molecular weight of approximately 17 kDa (VIP17) predicted to have four transmembrane domains and two extracellular loops. It is highly expressed in oligodendrocytes in the central nervous system, human T lymphocytes and kidney epithelial cells, cells that are targeted by Etx. MAL is also known to have four isoforms produced by alternative splicing [25,26,27].

Evidence to support MAL being a particularly attractive receptor candidate for Etx include: (1) MAL-knockout mice are resistant to Etx at doses at which wild type mice die rapidly [2]; (2) CHO cells and zebrafish endothelial cells that are normally resistant to Etx become susceptible to Etx when engineered to express human, rat or sheep MAL [2,24,28]; (3) Etx activity against oligodendrocytes, lymphocytes and endothelial cells is dependent on MAL expression [15,19,29]; and (4) Etx was found to interact with MAL in the cell membrane of lymphocytes by immunoprecipitation [15] and human erythrocytes by pulldown [17]. However, a direct interaction between Etx and MAL is yet to be demonstrated.

Alternative binding partners for Etx have also been proposed and include the extensively O-glycosylated hepatitis A virus cellular receptor 1 (HAVCR1) protein [30], caveolin-1 (CAV1) [28] and sulfatide [31]. Etx can also form functional pores in artificial lipid bilayers without the help of receptors, but with less efficiency [12,32,33].

Etx is a member of the aerolysin family of β-pore-forming toxins (aβ-PFTs) that are found in all kingdoms of life [34]. Like Etx, bacterial members of the aβ-PFTs often play key roles in disease, such as aerolysin produced by the human pathogen *Aeromonas hydrophila* [35], *Clostridium perfringens* enterotoxin (CPE) [36], and *Clostridium septicum* α-toxin [37]. Other bacterial members of the aβ-PFTs are non-pathogenic, such as *Bacillus thuringiensis* parasporin-2 [38] that kills human cancer cells, with potential applications in cancer therapy, while eukaryotic members of the aβ-PFTs often play a role in defence, such as lysenin produced by immune cells of the earthworm *Eisenia fetida* [39].

**Figure 1:**
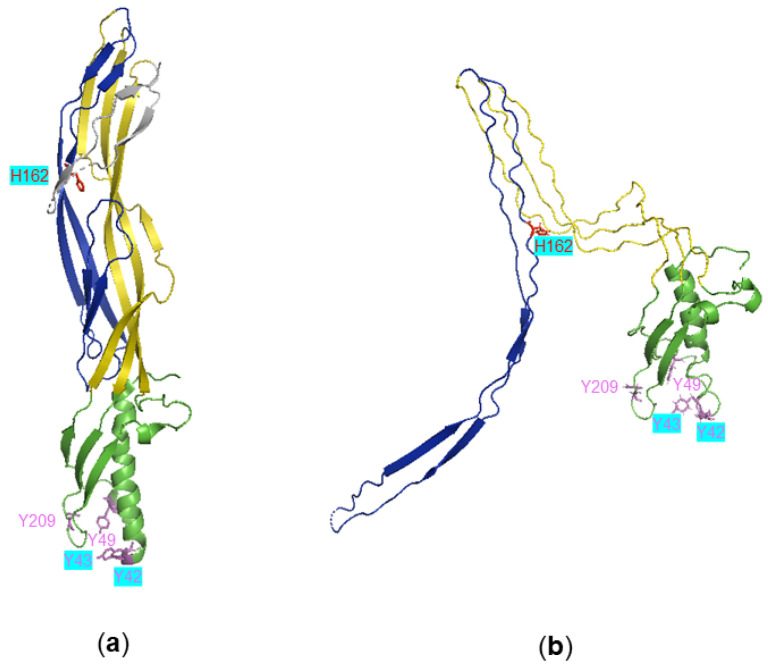
Structure of Etx before and after pore formation. Cartoon representation of monomeric Etx protoxin (PDB ID: 1UYJ) (**a**) and an Etx protomer from the heptameric pore structure (PDB ID: 6RB9) (**b**). Domains are coloured: receptor binding domain in green; β-hairpin domain in blue; cap domain in yellow. The carboxy terminal peptide is coloured in grey in Etx protoxin. Amino acids identified previously to be important in Etx binding (Y42, Y43, Y49, Y209) and toxicity (H162) are shown in stick representation; residues that are the focus of this study (Y42, Y43, H162) are highlighted in turquoise.

Crystal structures of the monomeric forms of aβ-PFTs reveal that members of the aβ-PFTs share a similar domain arrangement [34]: a receptor binding domain (RBD) that is unique to each member of the family and determines binding specificity, and probably explains why Etx is over 1000-fold more potent compared to other family members; and a structurally conserved pore forming module (PFM) (Figure 1, β-hairpin and cap domains) that contributes to the transmembrane β-barrel of the pore [34,40,41]. In some members of the aβ-PFTs, such as aerolysin and Etx, the PFM also contains a CTP, removal of which is critical for toxin activation [9] (Figure 1a).

The high-resolution structure of P-Etx monomer was solved in 2004 (PDB ID: 1UYJ) [42]. This revealed an elongated structure (100 Å × 20 Å × 20 Å) with a cluster of closely spaced surface exposed tyrosine residues (Y42, Y43, Y49 and Y209; Figure 1) in the amino-terminal RBD that has been implicated in receptor binding. In 2019 we have determined the 3.2 Å resolution cryo-electron microscopy structure of the Etx pore in MDCK cells (PDB ID: 6RB9) [43]. This revealed a mushroom-shaped complex, characteristic of most β-PFTs, that contains seven monomers. The heptameric pore is ~120 Å across, ~98 Å in height, with a pore size of ~24 Å in diameter. Each monomer in the pore structure contains three domains: the β-hairpin domain, the cap domain and the RBD (Figure 1b). The β-hairpin domain forms the inner β-barrel of the pore and the cap domain, which links the RBD to the β-hairpin domain at the extracellular side, includes the highly conserved double β-barrel (DBB) fold that is responsible for the SDS-resistant character of aerolysin pore [44] and may drive efficient pore formation [43]. 

Through site-directed mutagenesis studies we have previously identified tyrosine 43 (Y43) as a critical residue for Etx binding to MDCK cells [45]. However, our studies in ACHN cells, a human kidney cell line, and CHO cells engineered to express human MAL (CHO-hMAL) suggest that Y43 does not play a role in Etx binding to human cells, indicating that amino acids important for Etx binding differ between species [24,45].

Our pore structure of Etx in MDCK cells provides an explanation to the phenotype of important mutants, such as the reduced binding of Etx mutant Y43A to MDCK cells [45]; tyrosine 43 in the Etx pore is ideally located to interact with a receptor in the membrane [43]. The Etx pore structure also provides an explanation to the reduced cytotoxicity of Etx mutant H162A (H149A in [46]) towards MDCK cells; histidine 162 in the Etx pore forms a hydrogen bond with aspartate 250 in the cap domain that may stabilise the DBB fold and thus mutation H162A may affect efficient pore formation. Interestingly, Etx-H162A was completely inactive towards ACHN cells [45], indicating the complexity of Etx pore formation in different lipid environments.

In this study, we investigated the role of amino acids Y42, Y43 and H162, previously identified as important in Etx activity towards MDCK cells, in Etx activity towards CHO-hMAL cells, human red blood cells (hRBCs) and artificial lipid bilayers using site-directed mutants Etx-Y42A, Etx-Y43A and Etx-H162A, and a range of molecular, cell biology and biophysical approaches. We show that in CHO-hMAL cells Y42 is critical for Etx binding and not Y43 as in MDCK cells, indicating that surface exposed tyrosine residues in the RBD impact efficiency of Etx binding to MAL-expressing cells in a species-specific manner. We also show that Etx-H162A was unable to lyse CHO-hMAL cells but lysed hRBCs, whilst it was able to form pores in synthetic bilayers, providing substantial evidence of the complexity of Etx pore formation in different lipid environments. This will have implications in selecting a model system to assess the toxicity of Etx variants.

## 2. Results

### 2.1. Surface Exposed Tyrosine Residue Y42 in the RBD Is Critical for P-Etx Binding to CHO-hMAL Cells

**Figure 2:**
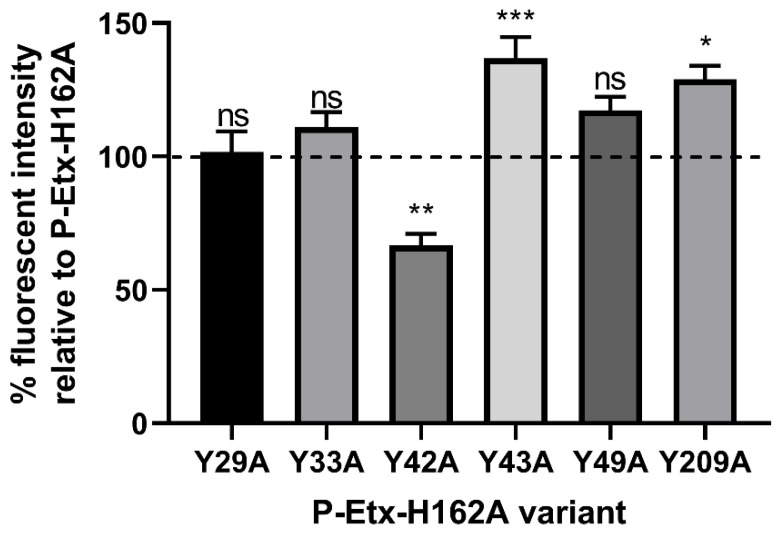
Effect of receptor binding domain mutations on the binding of P-Etx to live CHO cells expressing human MAL (CHO-hMAL). The binding of tyrosine mutants was assessed using On-Cell Western assay. Purified recombinant protoxins (5 µM) were added to wells containing CHO-hMAL cells. After incubation for 30 min at 37 °C, cells were fixed and bound protoxin was detected with rabbit anti-Etx and IRDye 800CW goat anti-rabbit antibodies. Fluorescence was imaged using an Odyssey infrared imaging system. The fluorescent signal from cells treated with P-Etx variants was normalized to that of cells treated with DPBS only and the binding activity of each tyrosine mutant was expressed as the percentage of fluorescence intensity relative to cells treated with P-Etx-H162A (100%), a mutant with preserved binding activity, using Image Studio ver. 5.2 software. To compare the means of On-Cell Western data, ordinary one-way ANOVA analysis followed by multiple comparisons test was carried out relative to P-Etx-H162A using the GraphPad Prism software v9 (GraphPad Software, La Jolla, CA, USA). Significant differences are indicated: *p* = 0.0124 (*), *p* = 0.0028 (**), *p* = 0.0007 (***), ns = not significant. Values represent the means of six independent experiments performed in triplicate.

In the first group of experiments we evaluated the three stages of Etx interaction (binding, oligomerisation and pore formation) with the plasma membrane of CHO-hMAL cells using molecular and cell biology approaches. We have previously generated site-directed mutants of P-Etx containing mutations Y16A, Y20A, Y29A, Y30A, Y36A or Y196A (Y29A, Y33A, Y42A, Y43A, Y49A or Y209A in this study) in H149A (H162A in this study) background to investigate the role of surface exposed tyrosine residues in the RBD in P-Etx binding to MDCK cells using On-Cell Western assay [45]. In the present study, we used the same set of P-Etx mutants and method to investigate the role of surface exposed tyrosine residues in the RBD in P-Etx binding to CHO-hMAL cells. As shown in Figure 2, P-Etx-H162A-Y42A showed significantly reduced binding to CHO-hMAL cells relative to P-Etx-H162A, while P-Etx-H162A-Y43A, identified in our previous study to be important in P-Etx binding to MDCK cells [45], showed increased rather than reduced binding to CHO-hMAL cells relative to P-Etx-H162A, indicating that amino acids important for Etx binding differ between species.

**Figure 3:**
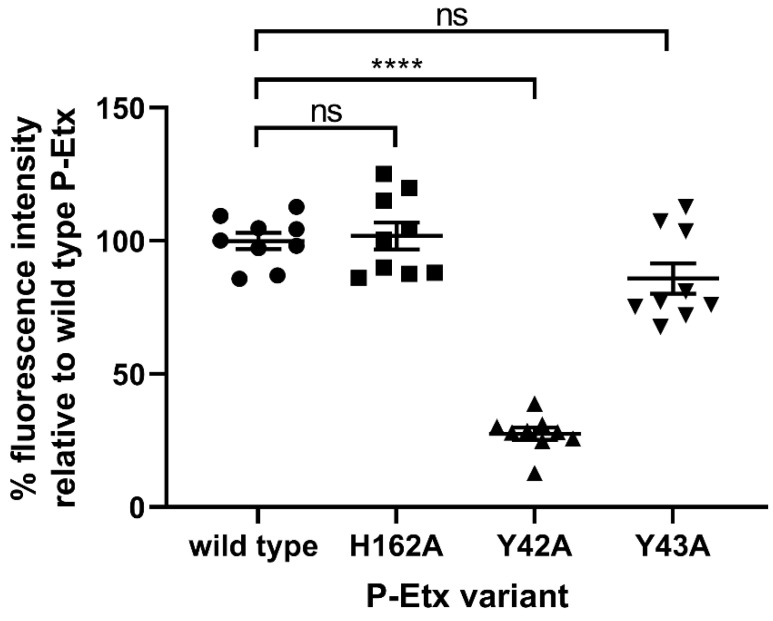
Effect of receptor binding domain and pore forming module mutations on the binding of P-Etx to live CHO-hMAL cells. The binding of Etx mutants to CHO-hMAL cells was assessed using On-Cell Western assay. Purified recombinant protoxins (5 µM) were added to wells containing CHO-hMAL cells. After incubation for 30 min at 37 °C, cells were fixed and bound protoxin was detected with rabbit anti-Etx and IRDye 800CW goat anti-rabbit antibodies. Fluorescence was imaged using an Odyssey infrared imaging system. The fluorescent signal from cells treated with P-Etx variants was normalized to that of cells treated with DPBS only and the binding activity of each P-Etx mutant was expressed as the percentage of fluorescence intensity relative to cells treated with wild type P-Etx (100%) using Image Studio ver. 5.2 software. To compare the means of On-Cell Western data, ordinary one-way ANOVA analysis followed by multiple comparisons test was carried out relative to wild type P-Etx using the GraphPad Prism software v9 (GraphPad Software, La Jolla, CA, USA). Asterisks denote a statistically significant difference (**** *p* < 0.0001). ns = not significant. Values represent the means of three independent experiments performed in triplicate.

To confirm that H162A mutation does not affect binding of P-Etx to CHO-hMAL cells, next we generated site-directed mutants P-Etx-Y42A, P-Etx-Y43A and P-Etx-H162A in wild type background. On-Cell Western assay shows that binding of P-Etx-H162A is similar to that of wild type P-Etx, and the binding pattern of P-Etx-Y42A and P-Etx-Y43A is similar to those of P-Etx-H162A-Y42A and P-Etx-H162A-Y43A (Figure 3). These data confirm that H162A mutation does not affect P-Etx binding to CHO-hMAL cells, and the key role of Y42 in P-Etx binding to CHO-hMAL cells.

### 2.2. Surface Exposed Tyrosine Residue Y43 Plays a Role in Oligomerisation of Etx in CHO-hMAL Cells

**Figure 4:**
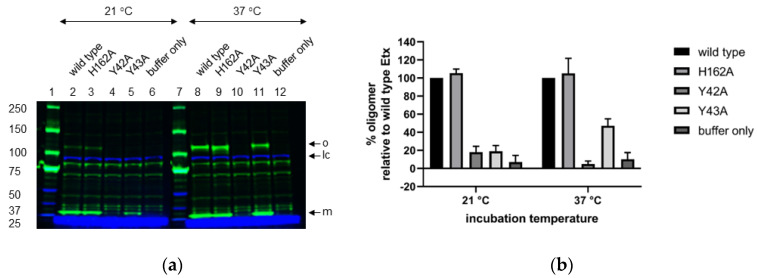
Western blot analysis of SDS-resistant Etx oligomers in CHO-hMAL cells. (**a**) CHO-hMAL cells were incubated with trypsin-activated Etx variants or buffer only at room temperature or 37 °C for 1 h. Following detergent solubilisation (1% (*w/v*) DDM), toxin complexes (30 µg per lane) were separated by SDS-PAGE and oligomerization was assessed by immunoblotting with anti-Etx polyclonal antibody. Lanes 1 and 7: Molecular weight marker; Lanes 2–6: CHO-hMAL cells incubated with trypsin-activated Etx-wild type, Etx-H162A, Etx-Y42A, Etx-Y43A or buffer only, respectively at room temperature; Lanes 8–12: CHO-hMAL cells incubated with trypsin-activated Etx-wild type, Etx-H162A, Etx-Y42A, Etx-Y43A or buffer only, respectively at 37 °C. Arrows indicate Etx monomer (m), Etx oligomer (o) and loading control (lc). Na^+^/K^+^-ATPase α1 (C464.6): sc-21712 antibody was used as loading control. One representative immunoblot out of three is shown for each experiment. The molecular masses (kDa) of protein standards are shown to the left of the blot. (**b**) Normalised, mean oligomer yields for Etx-H162A, Etx-Y42A and Etx-Y43A were determined by calculating the % oligomerisation relative to cells treated with wild type Etx (100%) using Image Studio ver. 5.2 software. Values represent the means of triplicate experiments.

Next, we evaluated the effect of Y42A, Y43A or H162A mutation on oligomerisation of trypsin-activated P-Etx in CHO-hMAL cells at room temperature and 37 °C by performing Western blot analysis of SDS-resistant oligomers (Figure 4 and Figure S1). Etx-H162A formed SDS-resistant oligomers similar to wild type Etx at both temperatures (Figure 4a; lanes 2–3 and 8–9). Etx-Y42A oligomers were hardly detectable at room temperature or 37 °C (Figure 4a; lanes 4 and 10, respectively) that correlates with reduced binding of P-Etx-Y42A to CHO-hMAL cells (Figure 2 and Figure 3). Etx-Y43A oligomers were hardly detectable at room temperature but oligomer yield recovered to ~40% relative to wild type Etx at 37 °C (Figure 4a; lanes 5 and 11, respectively), indicating that surface exposed tyrosine residue Y43 plays a role in oligomerisation of Etx in CHO-hMAL cells and oligomerisation of Etx-Y43A is temperature-dependent. Etx mutants formed SDS-resistant oligomers at the same apparent molecular weight as wild type Etx (~120 kDa).

### 2.3. Etx-H162A Is Inactive towards CHO-hMAL Cells

**Figure 5:**
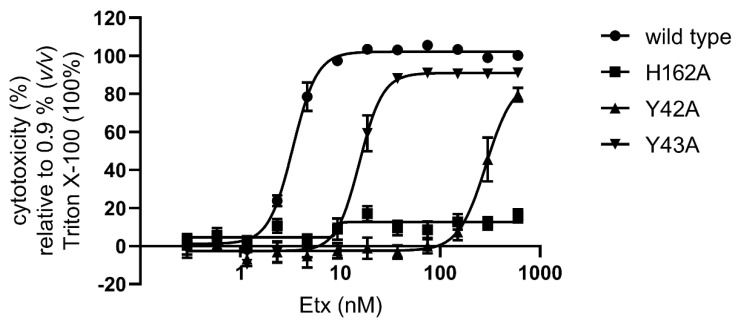
Effect of mutations Y42A, Y43A and H162A on the cytotoxicity of Etx towards CHO-hMAL cells. The cytotoxic activity of trypsin-activated Etx-wild type, Etx-H162A, Etx-Y42A or Etx-Y43A towards CHO-hMAL cells was determined by measuring the amount of LDH released into culture supernatants upon cell lysis (37 °C). Results were normalised to the signal from cells incubated with DPBS only (0% lysis) and cytotoxicity was expressed relative to cells treated with 0.9% (*v/v*) Triton X-100 (100% lysis). Values represent the means of three independent experiments performed in triplicate.

To evaluate the effect of Y42A, Y43A or H162A mutation on the cytotoxic activity of Etx towards CHO-hMAL cells we measured the amount of lactate dehydrogenase (LDH) released into culture supernatants upon cell lysis as described in Methods (Section 5.10). As shown in Figure 5, both tyrosine mutations resulted in reduced cytotoxic activity of Etx towards CHO-hMAL cells, indicated by an increase in the toxin dose required to kill 50% of the cell monolayer (CT_50_) relative to wild type Etx (Etx-wild type CT_50_ = 3.4 nM; Etx-Y42A CT_50_ = 287.8 nM; Etx-Y43A CT_50_ = 15.6 nM). Etx-H162A was unable to lyse CHO-hMAL cells despite its preserved binding (Figure 3) and oligomerisation (Figure 4) activities.

### 2.4. Haemolytic Activity of Etx-H162A Is Temperature Dependent, While Haemolytic Activity of Etx-Y42A Is Concentration Dependent

**Figure 6:**
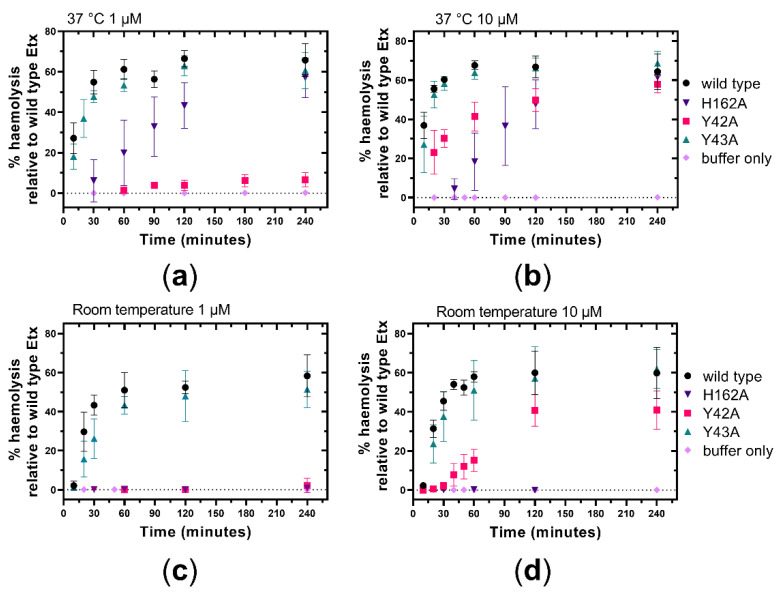
Effect of Y42A, Y43A or H162A mutations on the haemolytic activity of Etx towards hRBCs. hRBCs were incubated with trypsin-activated Etx variants or buffer only at room temperature (**c**,**d**) or 37 °C (**a**,**b**) at a dose of 1 µM (**a**,**c**) or 10 µM (**b**,**d**) for 4 h. Values represent the means of three independent experiments.

In a second group of experiments we investigated Etx mode of action in hRBCs. First, we evaluated the effect of Etx-Y42A, Etx-Y43A and Etx-H162A on the haemolytic activity of Etx towards hRBCs at room temperature and 37 °C using toxin concentrations 1 µM and 10 µM. Etx-Y42A was non-haemolytic at 1 µM (Figure 6a,c), while at 10 µM it became haemolytic at both temperatures (Figure 6b,d), indicating that haemolytic activity of Etx-Y42A is concentration dependent. After 1 h incubation, haemolytic activity of Etx-Y42A recovered quicker at 37 °C (approximately 40% haemolysis) compared to room temperature (approximately 14% haemolysis) (Figure 6b,d). Etx-Y43A showed no significant change in haemolytic activity relative to wild type Etx under any of the conditions tested (Figure 6a–d), indicating that in hRBCs Y43 does not play a role in Etx toxicity. Etx-H162A was inactive at room temperature at both concentrations tested (Figure 6c,d). However, its haemolytic activity recovered to wild type levels at 37 °C after 240 min of incubation (Figure 6a,b), indicating that haemolytic activity of Etx-H162A is temperature dependent.

### 2.5. Mutations Y42A and H162A Affect Oligomerisation of Etx in hRBCs

**Figure 7:**
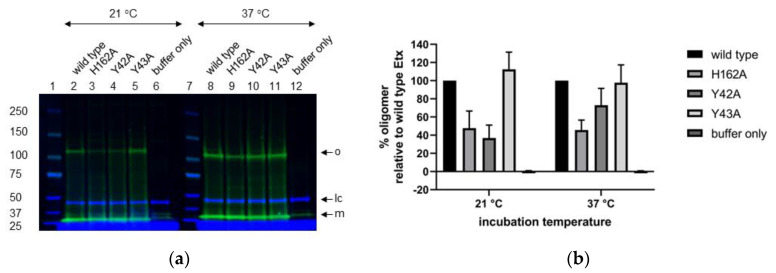
Western blot analysis of SDS-resistant Etx oligomers in hRBCs. (**a**) Human RBCs were incubated with trypsin-activated Etx variants or buffer only at room temperature or 37 °C for 1 h. Following detergent solubilisation, toxin complexes (30 µg per lane) were separated by SDS-PAGE and oligomerization was assessed by immunoblotting with anti-Etx polyclonal antibody. Lanes 1 and 7: Molecular weight marker; Lanes 2–6: hRBCs incubated with trypsin-activated Etx-wild type, Etx-H162A, Etx-Y42A, Etx-Y43A or buffer only, respectively at room temperature; Lanes 8–12: hRBCs incubated with trypsin-activated Etx-wild type, Etx-H162A, Etx-Y42A, Etx-Y43A or buffer only, respectively at 37 °C. Arrows indicate Etx monomer (m), Etx oligomer (o) and loading control (lc). FLOT2 antibody was used as loading control. One representative immunoblot out of three is shown for each experiment. The molecular masses (kDa) of protein standards are shown to the left of the blot. (**b**) Normalised, mean oligomer yields for Etx-H162A, Etx-Y42A and Etx-Y43A were determined by calculating the % oligomerisation relative to cells treated with wild type Etx (100%) using Image Studio ver. 5.2 software. Values represent the means of triplicate experiments.

Next, we evaluated the effect of mutations Y42A, Y43A or H162A on Etx oligomerisation in hRBCs at room temperature and 37 °C by performing Western blot analysis of SDS-resistant oligomers (Figure 7 and Figure S2). Oligomer yield of Etx-Y42A was reduced relative to wild type Etx at room temperature (approximately 37% compared to wild type Etx) but recovered at 37 °C (approximately 73% compared to wild type Etx) (Figure 7a; lanes 4 and 10) that correlates with increased haemolytic activity at 37 °C and 10 µM (Figure 6b,d). Oligomer yield of Etx-Y43A was similar to that of wild type Etx at both temperatures tested (Figure 7a; lanes 5 and 11) that correlates with preserved haemolytic activity under all conditions tested (Figure 6), indicating that Y43 does not play a role in Etx toxicity towards hRBCs. Oligomer yield of Etx-H162A was reduced relative to wild type Etx at both temperatures (approximately 50% compared to wild type Etx) (Figure 7a, lanes 3 and 9). Interestingly, despite being non-haemolytic at room temperature, Etx-H162A shows similar oligomer yields at both temperatures. Etx mutants formed oligomers at the same apparent molecular weight as wild type Etx (~120 kDa).

### 2.6. Effect of Etx on hRBC Morphology

**Figure 8:**
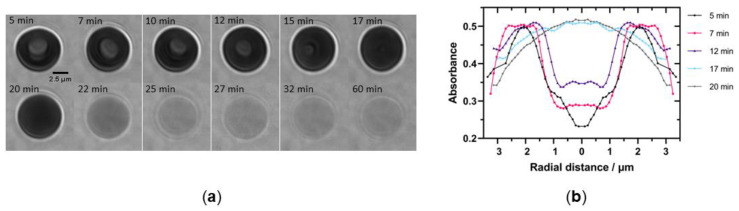
Effect of wild type Etx on hRBC morphology. (**a**) Images of a single cell between 5 and 60 min of incubation with 7 μM wild type Etx at room temperature. (**b**) Radially averaged variation of the optical absorbance at 415 nm at selected time points. To mimic the shape of hRBC, the radial absorbance profile of an individual cell was plotted and mirrored on the *y* axis.

In a separate group of experiments, we investigated the effect of Etx on hRBC morphology. As explained in Methods (Section 5.11), haemoglobin strongly absorbs light at 415 nm, which allows the reconstruction of cell shape from absorbance measurements. A healthy RBC forms a biconcave disc which appears on the absorbance image as a darker annulus with a brighter central region (Figure 8a). We recorded images of healthy hRBCs exposed to wild type Etx and followed changes in cell morphology. A typical example of a hRBC incubated with 7 μM wild type Etx at room temperature between 5 and 60 min after the addition of toxin is shown in Figure 8a. As can be seen from the images, initially the cell preserves its biconcave shape (5–12 min), but then gradually starts swelling and the dimple in the middle disappears (17 min). Figure 8b shows the radially averaged profiles at selected times. It can be clearly seen that the cell swells between 12 and 17 min. Haemolysis appears to take place shortly after the full swelling of the cell (17 min) and is manifested in Figure 8a by rapid loss of contrast between 20 and 22 min and later, due to loss of intracellular haemoglobin.

### 2.7. Electrophysiology of Etx Pores in Lipid Bilayers

**Figure 9:**
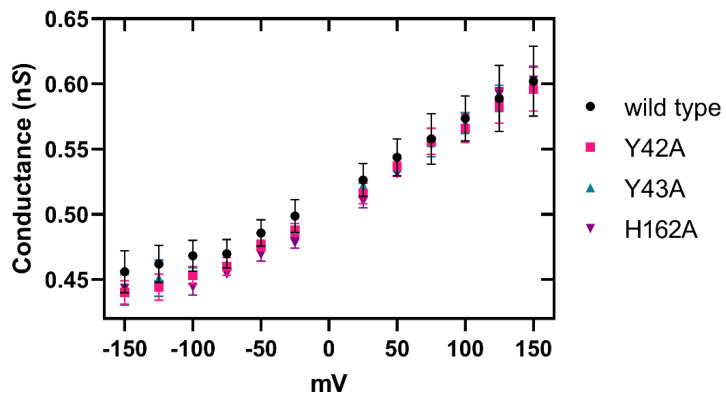
Electrophysiology of Etx pores in planar lipid bilayers. Average conductance of a single pore of each Etx variant over stepwise increase in voltage from −150 to 150 mV with standard deviation. Pores formed by spontaneous self-assembly in the presence of 0.1–0.8 µM activated Etx variants in the aqueous solution surrounding the lipid bilayer. Values represent the means of at least three independent experiments.

Finally, to investigate if RBD mutations Y42A and Y43A or PFM mutation H162A affect a hydrophobic-mediated pore formation mechanism by Etx in 1,2-diphytanoyl-sn-glycerophosphatidylcholine (DPhPC) lipid bilayers we employed the technique of single channel electrophysiology on black lipid membranes. The pore conductance for each applied voltage was calculated as described in Methods (Section 5.13) and there were insignificant differences in pore conductance of Etx mutants relative to wild type toxin over all voltages (Figure 9).

## 3. Discussion

We have previously provided key insights into the mechanisms by which Etx interfaces with the host cell membrane. Through site-directed mutagenesis studies we identified surface exposed tyrosine residue Y43 in the RBD of Etx as important for Etx binding to MDCK cells [45]. However, our more recent study suggested that Y43 does not play a role in Etx binding to CHO-hMAL cells [24]. Moreover, we have shown that Etx-H162A, previously reported to have 6-fold reduced cytotoxicity towards MDCK cells [46], was completely inactive towards the human kidney cell line ACHN [45], indicating the complexity of Etx pore formation in different lipid environments. In this study, we therefore investigated the role of amino acids Y42, Y43 and H162, previously identified as important in Etx activity towards MDCK cells, in Etx mode of action towards CHO-hMAL cells, hRBCs and artificial lipid bilayers using site-directed mutants Etx-Y42A, Etx-Y43A and Etx-H162A, and a range of molecular, cell biology and biophysical approaches.

Our cell binding studies revealed that in CHO-hMAL cells Y42 and not Y43, previously identified as important in Etx binding to MDCK cells, plays a critical role in Etx binding, suggesting that different tyrosine residues impact the efficiency of Etx binding to MAL-expressing cells in a species-specific manner. This exciting finding could explain the differences in potency and effect of Etx between species.

We hypothesised that if binding of Etx-Y42A was significantly reduced to CHO-hMAL cells, which correlated with severely impaired oligomerisation and cytotoxic activities, and if Etx binding to CHO-hMAL cells is dependent on the presence of the hMAL receptor, then activity of Etx-Y42A towards hRBCs would be severely impaired, too. However, Etx-Y42A was haemolytic towards hRBCs and was able to form oligomers, indicating that Etx mode of action in hRBCs differs from that in CHO-hMAL cells.

Haemolytic activity of Etx-Y42A increased with temperature at 10 µM that correlated with increased oligomer yield. The temperature increase appears to compensate the effect of the Y42A mutation; however, it does not repair it enough to match the activity of wild type toxin. This suggests that Y42A is defected in a way that the interaction with a receptor would be unstable, leading to transient binding and unbinding. Increased potency of Etx-Y42A at higher temperatures may suggest that more monomers are being bound to the membrane, resulting in increased receptor binding activity. Increased temperature could expose hydrophobic amino acids leading to enhanced hydrophobic binding efficiency, and/or expose receptor binding amino acids, which would enhance receptor binding. There is also a growing body of evidence for the importance of thermal shape fluctuations in regulating protein function such as dynamic contributions to allosteric regulation [47,48]. Thus, increased thermal fluctuation amplitudes may be a contributing factor for increased receptor binding of Etx-Y42A at higher temperatures.

Haemolytic activity of Etx-Y42A was also concentration dependent. Very little haemolysis was seen at 1 µM compared to 10 µM (Figure 6). This could be understood in terms of probability of binding, as the higher the number of molecules, the higher the likelihood of enough successful bindings to facilitate oligomerisation and therefore pore formation.

These results do not fully clarify whether Y42 is required for Etx binding to hRBCs but do provide evidence towards a complex interaction scenario where multiple binding mechanisms are used. It is possible that there is both hydrophobic-mediated and receptor-mediated interactions between Etx and the hRBC membrane. This hypothesis could explain the reduced haemolytic activity of Etx-Y42A relative to wild type toxin, where mutation of the Y42 amino acid prevents receptor-mediated but not hydrophobic-mediated binding, while Etx-wild type and Etx-Y43A may simultaneously employ both binding routes, and therefore have a more severe and rapid haemolytic affect. The Etx receptor identity on hRBCs remains controversial. A recent study suggested that MAL plays an important role in Etx-mediated haemolysis of hRBCs [17]. Other studies suggest that hRBCs do not express MAL and Etx indirectly causes haemolysis either by damaging neighbouring MAL-expressing T-lymphocytes [49] or by activating purinergic receptors (P2) [16].

Interestingly, despite the preserved binding of Etx-Y43A to CHO-hMAL cells, Etx-Y43A showed reduced oligomer yield relative to wild type Etx that correlated with reduced cytotoxicity. In contrast, oligomerisation and haemolytic activities of Etx were not affected by the Y43A mutation in hRBCs.

Our Etx pore structure assembled on MDCK cells has previously suggested that the reduced oligomer yield of Etx-H162A, which correlated with reduced cytotoxicity, may be due to reduced stability of the DBB fold, decreasing stability of formed oligomers and thus preventing efficient pore formation [43]. However, in hRBCs and CHO-hMAL cells Etx-H162A oligomer yield does not correlate with toxicity: in CHO-hMAL cells Etx-H162A showed oligomer yield similar to wild type toxin despite abolished cytotoxicity, while in hRBCs Etx-H162A showed similarly reduced oligomer yield at both temperatures despite Etx-H162A being non-haemolytic at room temperature and haemolytic at 37 °C. Therefore, haemolysis at the higher temperature cannot be explained by improved binding or oligomerisation mechanisms. This suggests that, specifically the pore formation step in hRBC membranes is affected by temperature. It is possible that a more fluid membrane at higher temperature would allow pore formation by Etx-H162A in hRBCs by a less stable DBB fold. However, we find that H162A was able to form pores in lipid bilayers at room temperature. It is unclear what temperature-dependent process, if any, could be responsible for facilitated pore formation by Etx-H162A in hRBCs at elevated temperatures, yet would allow pore formation in artificial DPhPC bilayers at room temperatures. One may speculate on the role of the mechanical properties of the bilayer membrane and the associated thermal fluctuations which could provoke pre-pore to pore transition. The bending elastic modulus of DPhPC bilayers at room temperature, 1.2 × 10^−19^ J [50] is much lower than that of hRBC membranes, 5.6 × 10^−19^ J [51], hence a DPhPC bilayer would exhibit thermal fluctuations with higher amplitudes, which could allow a successful pore formation. The restoration of pore formation seen in hRBCs at 37 °C may reflect increased membrane fluctuation amplitudes at this higher temperature.

The partially hydrophobic H162 amino acid is thought to be in an equivalent position to leucine 277 (L277) in aerolysin [44], and Etx mutant H162A has previously been equated to aerolysin mutant Y221G that oligomerises but does not form pores [52]. Both amino acids L277 and Y221 have previously been suggested to play a role in pore formation by preventing structural rearrangement of the β-hairpin domain [44], and H162 in Etx may therefore have a similar role. More recently the Etx pore structure revealed that H162 may also play a role in stabilising the DBB fold [43]. Interestingly, similar to MDCK cells [43], oligomer yield of Etx-H162A in hRBCs is reduced relative to wild type Etx, while in CHO-hMAL cells oligomer yield of Etx-H162A is similar to wild type, suggesting that in CHO-hMAL cells H162 affects unfolding of the β-hairpin domain but not the stability of the DBB fold.

Our electrical measurements on Etx mutants in DPhPC lipid bilayers reveal that their ion conductance capabilities are unchanged within the error of our measurements. This demonstrates that RBD mutations Y42A or Y43A, or PFM mutation H162A do not affect Etx pore formation in DPhPC lipid bilayers, in contrast to the complex variations observed in cells. Cells have many other structures on their membranes, such as the glycocalyx that would inhibit access to a protein. With most Etx-susceptible cells, a receptor is likely needed to overcome any barriers to binding. In addition, the heterogeneity of lipid species in the outer (and inner) leaflets of plasma membranes could affect monomer diffusion times and therefore oligomerisation time scales.

The radial profiles and images of hRBCs incubated with wild type Etx show cells swelling and becoming more rounded, followed by a loss of haemoglobin, resulting in empty ghosts (Figure 8a). Etx pores inner channels are reportedly only 24 Å in diameter [43] and allow molecules of up to 1 kDa in size to diffuse through [12]. As a single haemoglobin subunit is approximately 16 kDa, haemoglobin would not be able to leak through a pore, which suggests haemoglobin loss occurs due to a more significant disruption of the membrane. One study has shown that an Etx pore could activate P2 receptors causing Ca^2+^ influx which, in addition to the flux of K^+^ and Cl^-^ through the Etx pore, induces haemolysis [16]. P2 antagonists caused a large reduction of red cell lysis [16]. Another mechanism has since been suggested where Etx pores cause the cell to swell, leading to the activation of ICln chloride channels in order to reduce osmotic stress. These channels then become inhibited by the iron leaked through the pore as well as by copper leaked from nearby T-cells under attack [49]. The resulting lysis of the cell causes the haemoglobin leakage. The Etx-induced swelling of cells before lysis is also supported by previous work on MDCK cells [22].

## 4. Conclusions

In conclusion, our data provide substantial evidence of the complexity of Etx activity in different lipid environments and indicate that Etx toxicity in MDCK cells does not reflect Etx toxicity in human cells, which will have implications in assessing the toxicity of Etx vaccine candidates. Further research is needed to explore the role of thermal fluctuations in Etx pore formation.

## 5. Materials and Methods

### 5.1. Materials

All chemicals were purchased from Sigma, UK, unless otherwise stated. The polyclonal antibody against Etx mutant Y30A-Y196A (Y43A-Y209A in this study) was raised in rabbits as described in [53]. Protein concentrations were determined using the Pierce™ BCA Protein Assay Kit (Fisher Scientific Ltd., Loughborough, UK) and synthetic oligonucleotides were purchased from Eurofins Genomics, Germany.

### 5.2. Cell Culture 

Chinese hamster ovary (CHO) stable cell line expressing green fluorescent protein (GFP)-tagged human MAL (CHO-hMAL) was constructed and cultured as described previously [24]. In brief, CHO-hMAL cells were routinely cultured every 2 to 3 days in Dulbecco’s modified Eagle’s medium/Ham’s F12 medium (DMEM/F12, HEPES; Life Technologies, Carlsbad, CA, USA) with 2.5 mM L-glutamine, 15 mM HEPES, 0.5 mM sodium pyruvate and 1.2 g L^−1^ sodium bicarbonate supplemented with 0.05 mM nonessential amino acids and 10% foetal bovine serum Gold (PAA, Pasching, Austria) at 37 °C in a humidified atmosphere of 95% air / 5% CO_2_. In between culturing, cells were routinely detached by incubation in trypsin/EDTA and split as appropriate (typically 1:6 dilutions).

### 5.3. Cloning of Recombinant Epsilon Protoxin

The gene, *etxD*, encoding epsilon protoxin (P-Etx) with the H162A mutation (P-Etx-H162A) was subcloned from plasmid pCl0 into the expression vector pET-26b(+) (Merck, Darmstadt, Germany) using *Nco*I and *Xho*I restriction sites as described previously [45], which fused the amino-terminal end of P-Etx-H162A to the PelB leader peptide and the carboxy-terminal end of P-Etx-H162A to a polyhistidine (6 × His) affinity tag. Amino acid numbering corresponds to P-Etx with the 13 amino-terminal residues unless otherwise stated. Wild type recombinant P-Etx was produced and purified under ACDP/ACGM (Advisory Committee on Dangerous Pathogens/Advisory Committee on Genetic Modification) containment level 3 conditions.

### 5.4. Site-Directed Mutagenesis

Mutations were introduced into the gene encoding P-Etx using the QuickChange Lightning Site-Directed Mutagenesis Kit (Agilent Technologies Inc., Santa Clara, CA, USA) as described in [45]. The presence of the intended mutation was verified by DNA sequencing (Eurofins Genomics, Germany). 

### 5.5. Recombinant Protein Production and Purification

Recombinant P-Etx and its derivatives were expressed and purified in *E. coli* Rosetta 2 (DE3) cells (Merck, Darmstadt, Germany) as described in [45]. In brief, cells (100 mL) were grown in ZYM-5052-autoinducing medium supplemented with 50 μg mL^−1^ carbenicillin and 34 μg mL^−1^ chloramphenicol and cultured at 37 °C for 3 h at 300 rpm and at 20 °C for a further 24 h at 300 rpm. Cells were harvested by centrifugation (10,000*g* for 10 min at 4 °C) and the cell pellet was lysed by 10 mL BugBuster™ Protein Extraction Reagent (Merck, Darmstadt, Germany) containing rlysozyme™ (0.5 μL at 30 KU μL^−1^) (Merck, Darmstadt, Germany) and Benzonase^®^ Nuclease (10 μL at 25 U μL^−1^) (Merck, Darmstadt, Germany). The cell suspension was incubated on a rotating mixer for 25 min at room temperature and centrifuged (16,000*g* for 20 min at 4 °C) to separate soluble and insoluble fractions. The supernatant was loaded onto His GraviTrap columns (GE Healthcare Life Sciences, Little Chalfont, UK) and purification was carried out at 4 °C as described in [45]. In brief, His-tagged P-Etx was bound to the affinity column using a buffer containing 20 mM imidazole. The column was washed with a buffer containing 60 mM imidazole and recombinant P−Etx was eluted in a buffer containing 500 mM imidazole. For buffer exchange and sample clean-up, recombinant P-Etx was applied to a PD-10 Desalting Column (GE Healthcare Life Sciences, Little Chalfont, UK) following the manufacturer’s protocol and eluted in Dulbecco’s phosphate-buffered saline (DPBS), pH 7.0–7.2 (Invitrogen). 

### 5.6. Activation of P-Etx by Trypsin 

Purified recombinant P-Etx and its derivatives were activated with trypsin, TPCK (L-(tosylamido-2-phenyl) ethyl chloromethyl ketone) treated from bovine pancreas as described in [45]. In brief, trypsin was added to recombinant P-Etx at 1:100 (*w/w*) ratio and incubated at room temperature for 1 h. The reaction was stopped by adding trypsin inhibitor (0.66 mg per 1 mg trypsin) to the digest. 

### 5.7. Human Red Blood Cell Ghost Preparation

Human red blood cell ghosts were prepared as described previously [54,55]. In brief, 5 mL packed human red blood cells (Innovative Research) were washed three times in 45 mL DPBS, centrifuging cells (1500*g* for 10 min at 4 °C) between washes. Washed cells were resuspended in 50 mL DPBS (2.5 × 10^9^ cells/mL) and stored on ice for 30 min. After centrifuging cells (1500*g* for 10 min), the supernatant was removed, and hemolysis was performed by resuspending cells in 1:15 PBS (20 mOsm; pH 7.6). Cells were incubated for 30 min on ice and ghosts were pelleted at 20,000*g* for 40 min. Ghosts were washed three times in 20 mOsm buffer as above and resuspended in DPBS.

### 5.8. On-Cell Western Assay

On-Cell Western assay was used to measure binding of recombinant P-Etx and its derivatives to CHO cells expressing human MAL as described previously [45]. In brief, collagen-coated 96-well microtiter plates were seeded with 3.5 × 10^4^ cells/well in DMEM-F12 medium containing 10% foetal bovine serum (DMEM-F12/FBS) and plates were incubated overnight at 37 °C in a humidified atmosphere of 95% air / 5% CO_2_. The next day, cells were washed with DMEM-F12/FBS and incubated with purified recombinant P-Etx (5 µM) for 30 min at 37 °C in a humidified atmosphere of 95% air/5% CO_2_. For background control, cells were incubated with DPBS only. Unbound toxin was removed by washing cells three times with DMEM-F12/FBS. Cells were then fixed with 2% formaldehyde at room temperature for 15 min, washed with DPBS three times, and blocked for 1.5 h using Odyssey blocking buffer (LI-COR Biosciences, Lincoln, NE, USA). Bound P-Etx was detected with rabbit anti-Etx polyclonal primary antibody raised against recombinant P-Etx with Y43A and Y209A mutations [53] at 1:200 dilution and IRDye^®^ 800CW goat antirabbit IgG (H + L) secondary antibody (LI-COR Biosciences, Lincoln, NE, USA; catalogue number 926-32211) at 1:500 dilution. Fluorescence was detected using the Odyssey CLx infrared imaging system (LI-COR Biosciences, Lincoln, NE, USA). Quantitative analyses were performed using the Image Studio ver 5.2 software. 

### 5.9. Oligomerization Assay

Oligomerization assay was performed as described in [43], with modifications. In brief, CHO-hMAL cells were grown to confluence in 6-well plates (1.2 × 10^6^ cells per well), washed three times with DPBS to remove residual trypsin present in the cell culture medium, and incubated with 2 mL pre-warmed L15 medium containing trypsin-activated recombinant Etx-wild type, Etx-H162A, Etx-Y42A or Etx-Y43A (350 ng mL^−1^, equivalent to 1× CT_100_ dose of wild type Etx) at room temperature or 37 °C for 1 h to allow binding and oligomerization. Cells were subsequently washed three times with DPBS to remove unbound Etx, lysed by three cycles of freezing and thawing in 100 μL ice cold DPBS supplemented with Halt™ Protease Inhibitor Cocktail (100×) (Thermo Fisher Scientific, UK) at a final concentration of 1× and harvested by scraping. The collected cells were transferred to a microcentrifuge tube and solubilized in DPBS containing 1% (*w/v*) DDM by incubation at 4 °C overnight with occasional shaking. After incubation, insoluble material was removed by centrifugation (20,000*g* for 30 min at 4 °C) and the concentration of solubilized protein was determined by BCA assay.

Toxin complexes in solubilization buffer were analysed by SDS-PAGE and Western blot as described in [43]. In brief, solubilized proteins were resolved by NuPAGE™ 7% Tris-Acetate Protein Gels (Invitrogen Ltd., Paisley, UK) using Surelock Xcell apparatus (Invitrogen Ltd., Paisley, UK) using NuPAGE™ Tris-Acetate SDS Running Buffer (Invitrogen Ltd., Paisley, UK) and NuPAGE LDS sample buffer (Invitrogen Ltd., Paisley, UK) and gels were electrophoresed at 150 V for 1 h at room temperature. For SDS-PAGE the molecular weight standard Perfect Protein™ Marker, 10–225 kDa (Merck, Darmstadt, Germany) was used. After electrophoresis, proteins in the gel were transferred to a nitrocellulose membrane using the 15 min high molecular weight program of the Trans-Blot^®^ Turbo™ Transfer System (Bio-Rad). The membrane was blocked with PBS containing 0.1% Tween-20 and 5% skim milk and oligomeric complexes were detected with rabbit anti-Etx polyclonal primary antibody [53] and IRDye^®^ 800CW goat anti-rabbit IgG (H + L) secondary antibody (LI-COR Biosciences, Lincoln, NE, USA; catalogue number 926-32211) at 1:500 and 1:5000 dilutions, respectively. For loading control, the mouse monoclonal Na^+^/K^+^-ATPase α1 (C464.6): sc-21712 primary antibody (Santa Cruz Biotechnology, Dallas, TX, USA; catalogue number sc-21712) and the IRDye^®^ 680RD goat (polyclonal) anti-mouse IgG (H + L) secondary antibody (LI-COR Biosciences, Lincoln, NE, USA; catalogue number 925-68070) were used at 1:200 and 1:5000 dilutions, respectively. Antibodies were diluted in PBS containing 0.1% Tween-20 and 3% skim milk. Immuno-reactive bands were detected using the Odyssey CLx infrared imaging system (LI-COR Biosciences, Lincoln, NE, USA). Quantitative analyses were performed using the Image Studio ver 5.2 software and the Housekeeping Protein Normalization Protocol (LI-COR Biosciences, Lincoln, NE, USA). 

Human RBC ghosts (20 µg total protein) were incubated with trypsin-activated Etx variants (10 µM final concentration) or buffer only at room temperature or 37 °C for 1 h. Following detergent solubilisation, toxin complexes (30 µg per lane) were separated by SDS-PAGE and oligomerization was assessed by immunoblotting with anti-Etx polyclonal antibody as described above. For loading control, the mouse monoclonal Flotillin 2 (FLOT2) antibody (Proteintech, Rosemont, IL, USA; catalogue number 66881-11g, lot 10008101, Genbank Accession no. BC017292) and the IRDye^®^ 680RD goat (polyclonal) anti-mouse IgG (H + L) secondary antibody (LI-COR Biosciences, Lincoln, NE, USA; catalogue number 925-68070) were used at 1:2000 and 1:5000 dilutions, respectively.

### 5.10. Cytotoxicity Assay

The cytotoxic activity of trypsin-activated Etx and its derivatives toward CHO-hMAL cells was determined by measuring the amount of lactate dehydrogenase (LDH) released into culture supernatants upon cell lysis using the CytoTox 96 Nonradioactive Cytotoxicity Assay Kit (Promega, Southampton, UK) as described in [24]. In brief, a 2-fold dilution series of each trypsin-activated toxin (ranging from 600–0.292 nM) was prepared in DPBS and added to cells seeded into 96-well plates (3 × 10^4^ cells per well), alongside negative (DPBS) and positive (0.9% (*v/v*) Triton X-100) controls. After incubation at 37 °C for 3 h, cell culture medium (50 µL) was transferred to an empty 96-well plate and reconstituted substrate mix (50 µL) was added to each well. The plate was incubated for 30 min at room temperature in the dark. Absorbance at 490 nm was measured using a Tecan Infinite 200 PRO Microplate Reader (Tecan Group Ltd., Männedorf, Switzerland). Results were normalised to the signal from cells incubated with DPBS only (0% lysis) and cytotoxicity was expressed relative to cells treated with 0.9% (*v/v*) Triton X-100 (100% lysis). The toxin dose required to kill 50% of the cell monolayer (CT_50_) was determined by nonlinear regression analysis, fitting a variable slope log (dose) vs. response curve, constraining parameter *F* (response *F* percent) to a value of 50, $\log\left(CT_{50}\right)=\log\left(CTF\right)-\left(1/HS\right)\times \log\left[F/\left(100-F\right)\right]$, where *CTF* is the concentration of Etx that gives a response 50 percent of the way between Bottom and Top, which are plateaus in the units of the Y axis, *HS* is the hill slope and *F* is the percent response.

### 5.11. Erythrocyte Morphology

We used optical absorption measurements of individual cells to monitor the effect of Etx on hRBC morphology. Images of individual cells were captured in transmission mode using a 415 nm diode as a light source (where haemoglobin has a high absorption) on an Olympus IX50 inverted microscope (Olympus Optical, Hamburg, Germany) equipped with an Olympus UPlanSApo 60×/1.20 W objective, an AVT Stingray F-145B camera and Live Acquisition 2.2.0.8 software. In the first approximation, optical absorbance is linearly proportional to the cell thickness, which allows for the reconstruction of the cell shape from the measured optical absorbance across the image of the cell. Radially averaged absorbance profiles were calculated using a custom Python script as described in [56].

### 5.12. Haemolysis Assay 

Blood from healthy volunteers was taken using a finger prick lancet and added to a solution of BSA/PBS, to which Etx was gently mixed in. The final sample concentration was 10 µM Etx, 0.5% blood, and 0.1% BSA in PBS. As soon as Etx was added the sample was immediately injected into a microscope slide chamber. Images were taken with an AVT Stingray F-145B camera every two to five minutes using an inverted microscope (Olympus IX50, Olympus Optical, Hamburg, Germany), 60× water objective (UPlanSApo 1.20 W, ∞/0.13–0.21/FN26.5, Olympus), and an ultraviolet (UV) light source at 415 nm (ThorLabs). The UV light was turned off between images to limit any radiative damage to the cells.

To conduct the heated experiments, a stage top incubator with a slidable lid and glass plates were used (Heating Incubation Insert P-set 2000, Petri dishes “35” POC-R + mini, PeCon GmbH, Erbach, Germany) with a digital temperature controller unit (tempcontrol 37-2 digital, PeCon GmbH, Erbach, Germany). As only one microscope slide can fit inside the incubator, a slide with multiple wells (Ibidi 18 well µ-slide) was used. 30 µL of each sample mixture was transferred to individual wells on the slide. The slide was immediately placed inside the stage top incubator which had been pre-heated to 37 °C. At this point the temperature of the objective was set to 37 °C using an attached objective heater, to limit heat loss through contact with the objective.

For all hemolysis experiments, five images from different areas of the slide were taken, per measurement, to reduce variation due to any uneven distribution of cells on the slide. The number of ghost cells were counted by eye and the number of viable cells were counted using a Python script. The percentage of ghost cells were calculated, averaged, and plotted against time to create the lysis curves.

### 5.13. Electrophysiology 

A 1,2-diphytanoyl-sn-glycero-3-phosphocholine (DPhPC) lipid bilayer was formed by opposing two lipid monolayers, as described previously [57], over a 50–100 µm hole in a PTFE film separating two water filled compartments. A 2% hexadecane solution was applied to the dry PTFE film to aid adherence of lipids. Each side of the cuvette was filled with 850 µL of an aqueous buffer (1 M KCl, 5 mM MES, pH 6). Initially, 5 µL Etx (0.5 mg/mL) was added to one side of the cuvette and the solution was mixed by pipetting up and down. More Etx was added if a pore did not form within a reasonable time. Final Etx concentrations ranged between 0.1 and 0.8 µM (5–40 µL of 0.5 mg/mL Etx stock). Experiments were conducted at room temperature. Pore conductance, *G*, was calculated as G=I/V, where *I* is the current and *V* the voltage.

### 5.14. Statistics 

Data were analysed using the GraphPad Prism software v9 (GraphPad Software Inc., La Jolla, CA, USA). Error bars are the standard error of the means (±S.E.M.) unless otherwise stated. 

## Data Availability

The data presented in this study are available on request from the corresponding authors.

## Supplementary Materials

The following supporting information can be downloaded at https://www.mdpi.com/article/10.3390/toxins14110757/s1, Figure S1: Western blot analysis of SDS-resistant Etx oligomers in CHO-hMAL cells; Figure S2: Western blot analysis of SDS-resistant Etx oligomers in hRBCs.
