# Supplementary material for: Interaction of Clostridium perfringens Epsilon Toxin with the Plasma Membrane: The Role of Amino Acids Y42, Y43 and H162
Source: Toxins (Basel). 2022 Nov 3;14(11):757. doi: 10.3390/toxins14110757 (PMC9694948; doi:10.3390/toxins14110757)
Supplement: Supplementary file 1 [file toxins-14-00757-s001.zip › toxins-1964088-supplementary.pdf]

toxins

Supplementary Materials: Interaction of *Clostridium perfringens* Epsilon Toxin with The Plasma Membrane: The Role of Amino Acids Y42, Y43 and H162

Skye Marshall, Beth McGill, Helen Morcrette, C. Peter Winlove, Catalin Chimerel, Peter G. Petrov and Monika Bokori-Brown

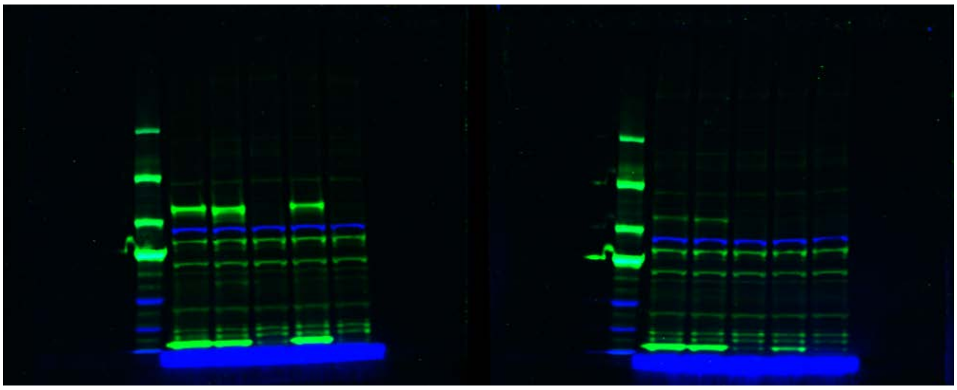

Figure S1. Western blot analysis of SDS-resistant Etx oligomers in CHO-hMAL cells.

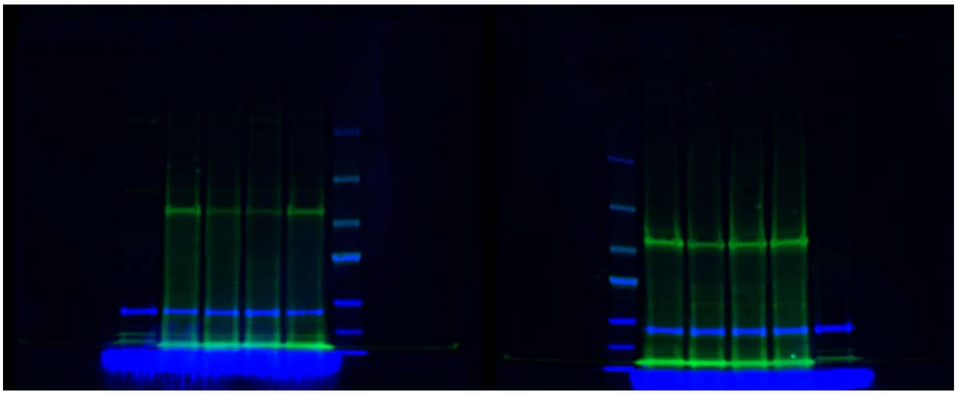

Figure S2. Western blot analysis of SDS-resistant Etx oligomers in hRBCs.
